# Carotid Artery Stenting 2013: Thumbs up

**DOI:** 10.4021/cr253w

**Published:** 2013-03-08

**Authors:** Philipp Wagdi

**Affiliations:** Interventional Cardiology, HerzZentrum Hirslanden, Witellikerstrasse 36, 8008 Zurich, Switzerland. Email: wagdi@herzzentrum.ch

**Keywords:** Carotid artery stenting, Carotid endarterectomy, Stroke, Carotid artery stenosis

## Abstract

It has been customary for interventional cardiologists involved in carotid artery stenting, to underline non-inferiority of the percutaneous technique versus surgical carotid endarterectomy. To that end, all cause morbidity and mortality figures of both methods are compared. Surgery has, in most large randomized studies, had an edge over stenting in terms of cerebrovascular adverse events. This may have partly been due to occasional indiscriminate indication for stenting in lesions and/or vessels with unfavourable characteristics (severe target vessel tortuosity and calcification, Type III aortic arch, and so on). On one hand, the author pleads for improvement of the excellent results of endarterectomy, by subjecting all patients planned for surgery to a thorough preoperative cardiological work up, including generous invasive investigation, thus reducing the incidence of perioperative myocardial infarction, heart failure and cardiac death. On the other hand, we are convinced that the results of carotid stenting should then be compared to best practice surgery. The rate of neurological adverse event rate after carotid endarterectomy at our institution lies under 0.7% at 30 days postoperatively. Specifically, the goal should be that carotid stenting underbids surgical endarterectomy, also and mainly, in terms of cerebral and cerebrovascular adverse events. Cardiac morbidity and mortality as well as laryngeal nerve palsy should no more be the main arguments for the percutaneous approach. This should easily be possible if patient selection for carotid revascularisation would be approached according to morphological criteria, in analogy with the “Syntax”-score used to optimise revascularisation strategies in coronary artery disease.

## Introduction

Carotid endarterectomy (CEA) is still considered the gold standard in treatment of both symptomatic and asymptomatic carotid artery stenosis. Evidence based criteria and guidelines [1-4] should remain the mainstay for every argumentation pro or contra any diagnostic or therapeutic procedure. Yet, even guidelines may sometimes be subject to “party politics”. On the other hand, it may be difficult to command an exhaustive overview of a particular issue, so that opinion and decision making will necessarily contain some “best guess” ingredient. Widespread opinion, especially among neurologists, is that carotid artery stenting (CAS) is inferior to CEA in terms of adverse events and long term outcome. There may be a part of self-incurred responsibility by interventionalists to the reputation of the procedure because of indiscrimate and uncritical application of CAS.

Dogmatic positions are then, at best, based on accumulated (peri-) institutional experience, or, at worst, on biased personal particular interest. It is high time that recommendations for revascularisation-treatment of carotid artery lesions be issued independent from personal interest, party politics or best guesses. Interventionalists should not only argue with superiority of CAS in terms of cardiac mortality and morbidity. The goal of CAS should be to achieve at least non-inferiority in terms of long term follow up related to cerebrovascular disease (stroke, transient ischemic attacks, cerebral cognitive changes, and so on).

This goal should be achieved primarily by setting the bar higher for the interventional cardiologist. The aim is to improve the reputation of CAS, by reducing both qualified as well as unqualified prejudice to the method. A four point approach would be: 1). To reduce morbidity and mortality of surgical (CEA) by systematic cardiological screening and treatment of patients (and this is not a paradox, but in the very interest of all carotid revascularisation techniques including CAS); 2). To adhere to a comprehensive periprocedural risk stratification by the individual interventionalist before indicating CAS; 3). To tackle carotid lesions interventionally only after comprehensive assessment of clinical and vessel/lesion variables, and weighing them up against the interventionalist’s background, experience and skills; 4). To compare CAS and CEA based solely on procedural performance in terms of stroke, transient ischemic attacks, cerebral cognitive changes and target lesion failure.

**Figure 1:**
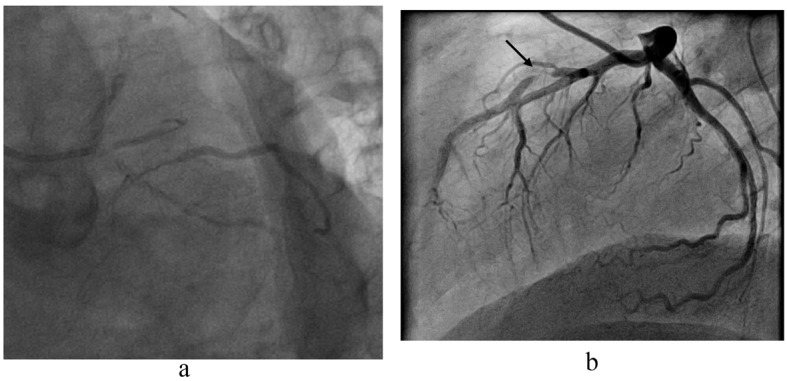
(a). Common sense analogy from coronary artery revascularisation strategies. Not many sane interventional cardiologists would advocate primary percutaneous treatment for this symptomatic severe left main, left anterior descending, circumflex and intermediate coronary lesion with a collateralised periphery from the right coronary artery. On the other hand, only a few cardiac surgeons would seriously advise bypass grafting as first line treatment for the focal, single vessel lesion depicted in (b).

In analogy to the Syntax-score [5] worked out as a tool for grading the complexity of coronary artery disease and ultimately suggesting a framework for the choice of the revascularisation technique used for the individual patient, we think that carotid artery disease should eventually undergo a similar consensual evaluation (Fig. 1).

## It is in the Interest of Interventionalists to “Set the Bar Higher” for CAS

Although there is a general feeling among vascular surgeons, neurologists, interventional cardiologists and neuroradiologists, that the subject is controversial, closer scrutiny shows that data comparing CAS to CEA seem to be quite consensual. Whereas all cause mortality and morbidity may more or less be equal, rates of stroke and transient ischemic attacks seem to be slightly higher in patients undergoing CAS than CEA [1-4].

For the time being, it cannot realistically be expected that the relatively acceptable adverse event rates reported in randomized multicenter studies can be reproduced in all real world centers performing CAS and CEA, although such results have been published [6]. Common sense would suggest that the good results of CEA (4 year rate of death, periprocedural or ipsilateral stroke, myocardial infarction 6.8% in CREST) could even be bettered by involving a knowledgeable cardiologist and internist in preoperative workup. We propose that every patient presenting with carotid artery disease requiring revascularisation should undergo either a conclusive imaging modality to exclude ischemia or coronary angiography to exclude the presence of prognostically relevant coronary artery disease. This measure would reduce the incidence of a relevant cardiac morbidity and/or mortality in patients undergoing CEA, and thus further improve results of CEA, thus indirectly setting a higher bar for interventional cardiologists and CAS. At our institution, a total of 493 CEAs were performed by three cardiac and vascular surgeons from 2003 to 2012 (the following data were kindly provided by Dr. A. Laske, see acknowledgements). Of these, 339 were isolated CEAs and 154 CEAs combined with coronary bypass and/or valvular surgery in the same procedure. Thirty day follow up of the patients undergoing isolated CEAs (the population we seek to set as reference) revealed 0.6% neurological adverse events (2 patients suffered an ipsilateral stroke) and no mortality. Despite the high flying goal, the author firmly believes that CAS should not only be superior to CEA in terms of overall mortality and morbidity, but also at least non-inferior in terms of long term follow up related to cerebrovascular disease (stroke, transient ischemic attacks, cerebral cognitive changes, and so on). For the time being, data may suggest slight superiority of CEA over CAS when only neurological outcome is examined [1-3]. In order to rectify this skewness, interventionalists should exert short term constraint in patient selection. For example it is well known that some vessel and/or lesion characteristics [7-9] are accompagnied by a significantly higher risk for CAS. If diagnostic workup indicates high pre-intervention risk, then the involved interventionalist should first review the situation, reassessment being based on his experience, skills and previous record.

Using lesion morphology or patient morbidity as a defensive or post hoc argument in case of adverse event is unethical. One of the significant practical aspects contributing to a safe approach is to have an invasive appreciation of aortic morphology and pathology (tortuosity, calcification, dimensions), as well as target vessel and lesion characteristics. The author has more than once aborted a procedure, after diagnostic catheterization showed very unfavourable morphology, unrevealed by ultrasound or magnetic resonance imaging. This policy may have negative short term repercussions on income and case load for the individual interventionalist. The positive long term aspects are however incomparably more gratifying, and not to mention patient safety and ethical issues.

## Specific Target Vessel and Lesion Aspects (Table 1)

There are some access and target vessel features that render CAS not only more challenging technically, but also bear a significantly higher periinterventional adverse event risk. It has been customary to consider a visible (fresh) thrombus in the lesion as a contraindication to CAS. The lesion can generally be tackled after resolution of thrombus under energetic anticoagulation, antiplatelet and lipid lowering medication for 2 - 3 months.

**Figure 3:**
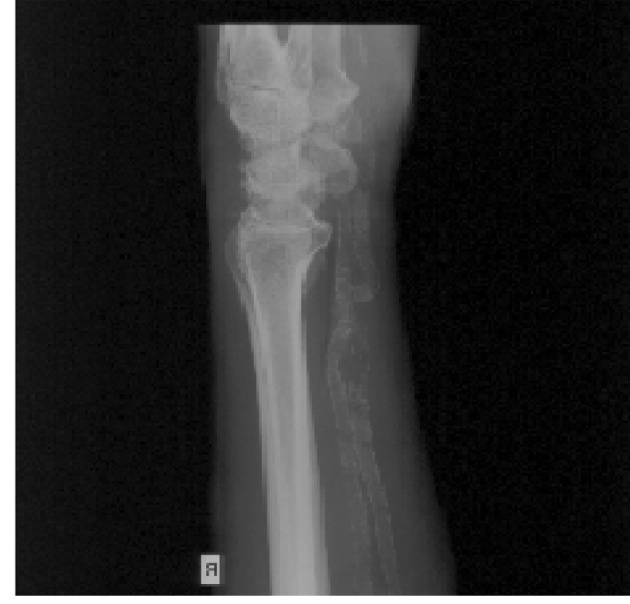
Severe calcification of the radial and ulnar artery in an 81-year-old patient with patent radial and ulnar pulse, precluding carotid or coronary intervention using radial access.

We have found contralateral occlusion per se, not to be a major impediment to safe CAS (10), concomitant pathology has however to be looked at. Type III aortic arch is defined as elongation and rostral migration of the arch, with the brachial artery trunk originating lower than the left subclavian artery empirically by more than 2 diameters of the left common carotid artery. Type III arch is technically challenging because of the difficulty in achieving a stable vessel intubation and backup. The radial approach has been advocated as an alternative [11, 12], but the approach may sometimes not be successful and may have to be abandoned for a femoral access [11]. Rarely the radial artery is severely calcified, precluding an intervention (Fig. 3).

**Figure 2:**
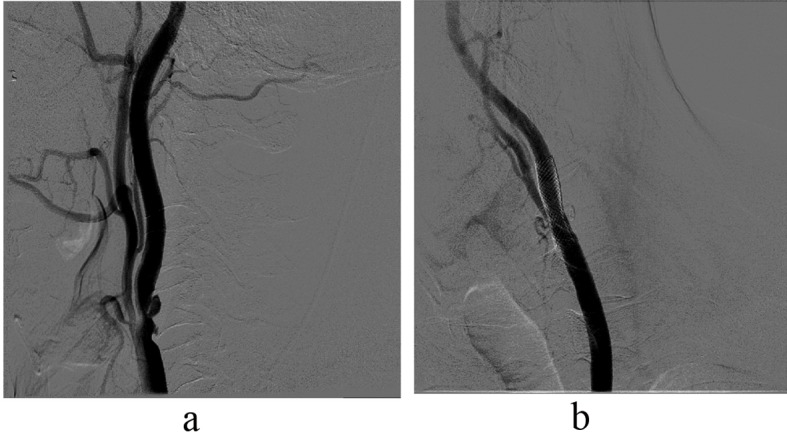
(a). Ulcerated plaque in a 62-year-old patient with contralateral complete chronic occlusion of the internal carotid artery. (b). Result after stenting. The patient is event- and symptom-free, three years after the intervention.

A left common carotid artery arising from the brachiocephalic trunk can impede adequate cannulation of the vessel and catheter stability when attempting a femoral approach, especially if the aorta is tortuous. Here again, a right radial approach might be helpful. Relevant calcification of target left common or internal carotid lesion is, especially if associated with tortuosity of the target artery either proximal or distal to the target lesion is in the author’s view one of the major predictors of cerebral adverse events and should rarely be attempted if CEA is a valid option. An ulcerated calcified plaque on top of a stenosis (Fig. 2) may also represent a certain risk, and care is warranted, especially if post-dilatation is contemplated. Here predilatation should clearly be avoided whenever feasible. Severe aortic arch calcification is a relevant risk for adverse cerebrovascular events when catheter manipulation is not straightforward, especially if the target vessel is tortuous, or a Type III arch is present. If femoral approach is chosen, then calcification of the arch can be a serious risk when intubation and catheter stability in the case of a left common carotid originating from the brachiocephalic trunk is difficult. The same applies to tortuous aortic arch, descending thoracic aorta and abdominal aorta.

CAS should in our opinion be preceded, then indefinitely followed by compliant and adequate lipid lowering and antiplatelet treatment. This medication should be maintained indefinitely.

Obviously adverse event risk is minimized by avoiding unnecessary interventions. Asymptomatic lesions < 70% (although often categorized as being low risk) need not to be treated. Much more useful in these cases is rigorous lipid lowering and platelet-inhibiting medication, aiming at stabilization of the plaque-cap.

**Figure 4:**
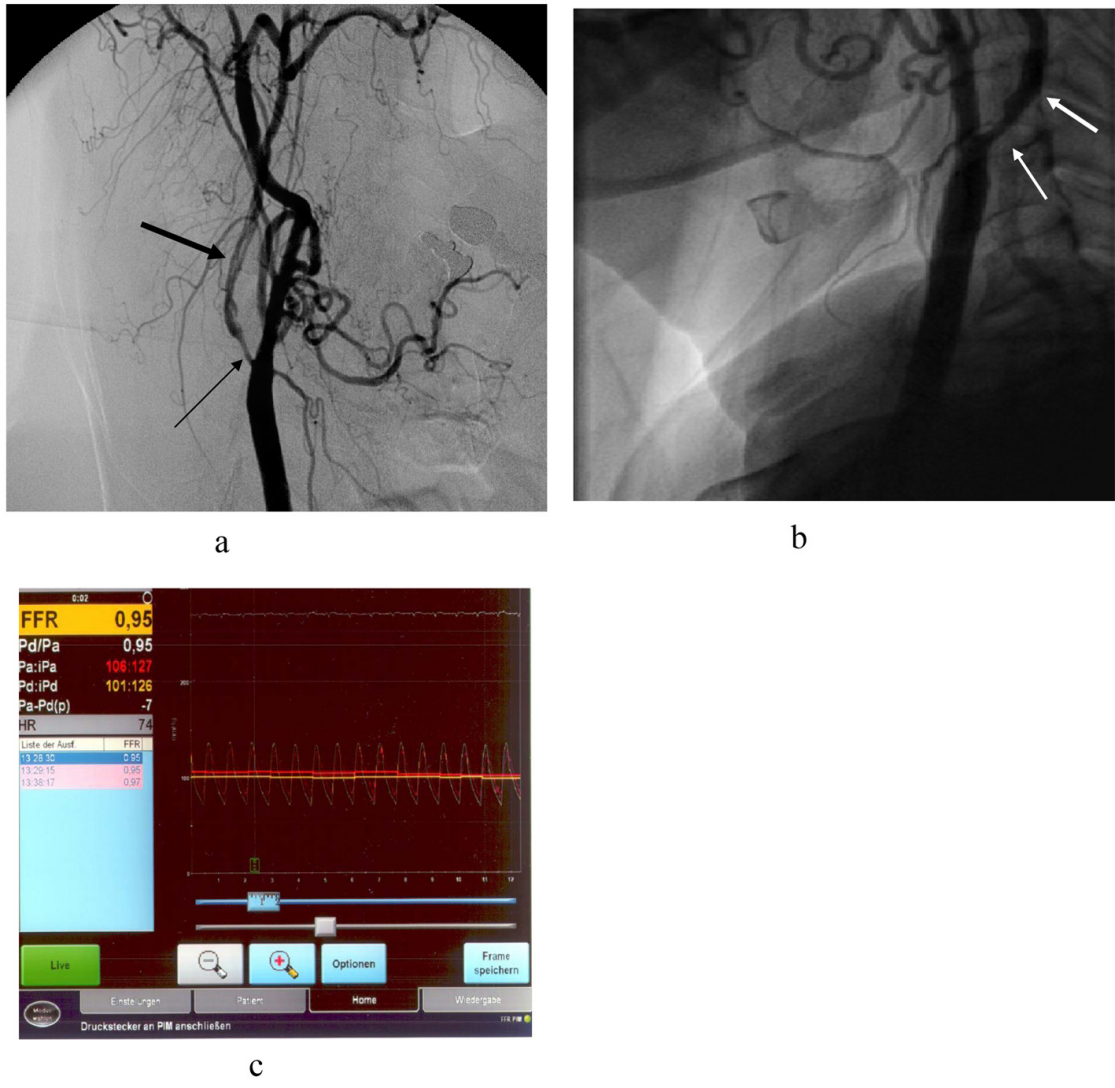
(a). Symptomatic subacute dissection (thick arrow) of the right internal carotid (thin arrow) in a 64-year-old woman; (b). Repeat angiography one and a half year later, indicated after sonographic diagnosis of severe restenosis, shows only moderate intimal hyperplasia (arrow) and restenosis; (c). Pressure gradient (0.95) measurement across the lesion confirms good functional result. The patient is discharged without undergoing the planned PTA and drug eluting balloon treatment.

Duplex ultrasound may grossly overestimate restenosis after CAS [13]. After performing diagnostic angiography, if the lesion seems not to be significant visually and by quantitative analysis, functional testing (for example pressure wire probing) can be helpful (Fig. 4), although angiographic morphological analysis is often enough for decision making.

## Outlook

**Figure 5:**
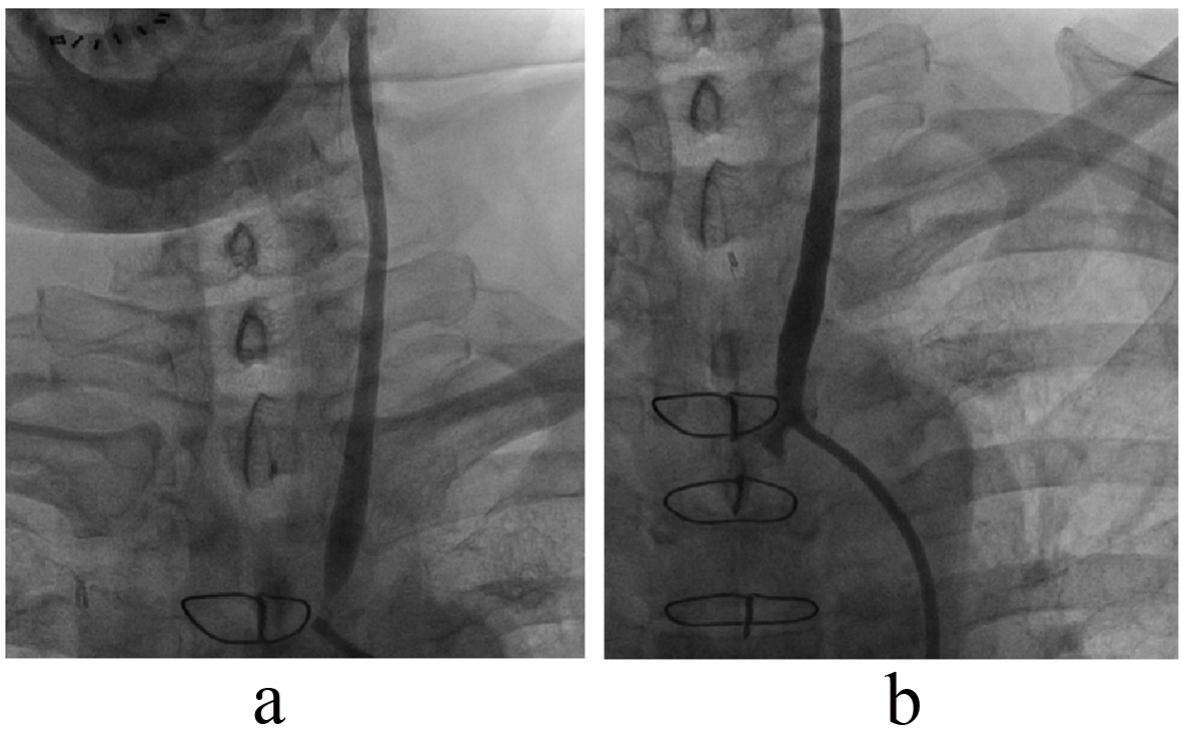
(a). Severe ostial left common carotid stenosis in a 60-year-old patient, heavy smoker, with severe chronic obstructive lung disease and bypass surgery 8 days previously. (b). Result after balloon-expandable stent implantation.

Since 1994, when CAS was first introduced for treatment of carotid artery stenosis, most neurologists have been pro CEA. Referral to CAS, at least in our institution, has been limited to cases not readily amenable to CEA, for example for critical lesions of the left common carotid at the ostium (Fig. 5). As a consequence, the author has proposed CAS mainly to his own patients undergoing diagnostic invasive cardiac workup. Obviously this leads to a relevant reduction of case load, the author has been performing an average of 6 to 10 selected cases per year for the last 4 years. But again, this seems to be a generalized problem [6]. In one of the “real world practice” communications, 38 persons in 6 centers performed 430 CAS cases over a period of 8 years [6], leading to an average of less than 10 cases per year per center. The average of cases per operator per year cannot be meaningfully extrapolated from this data. The same communication reports that 7,649 cases of CEA have been performed over the same time span by 111 surgeons in 6 institutions, yielding an average of 159 cases per center per year or about 9 cases per surgeon per year. In our institution, 3 surgeons performed a total of 493 CEAs over 10 years, yielding an average of 16 CEAs/surgeon/year.

**Figure 6:**
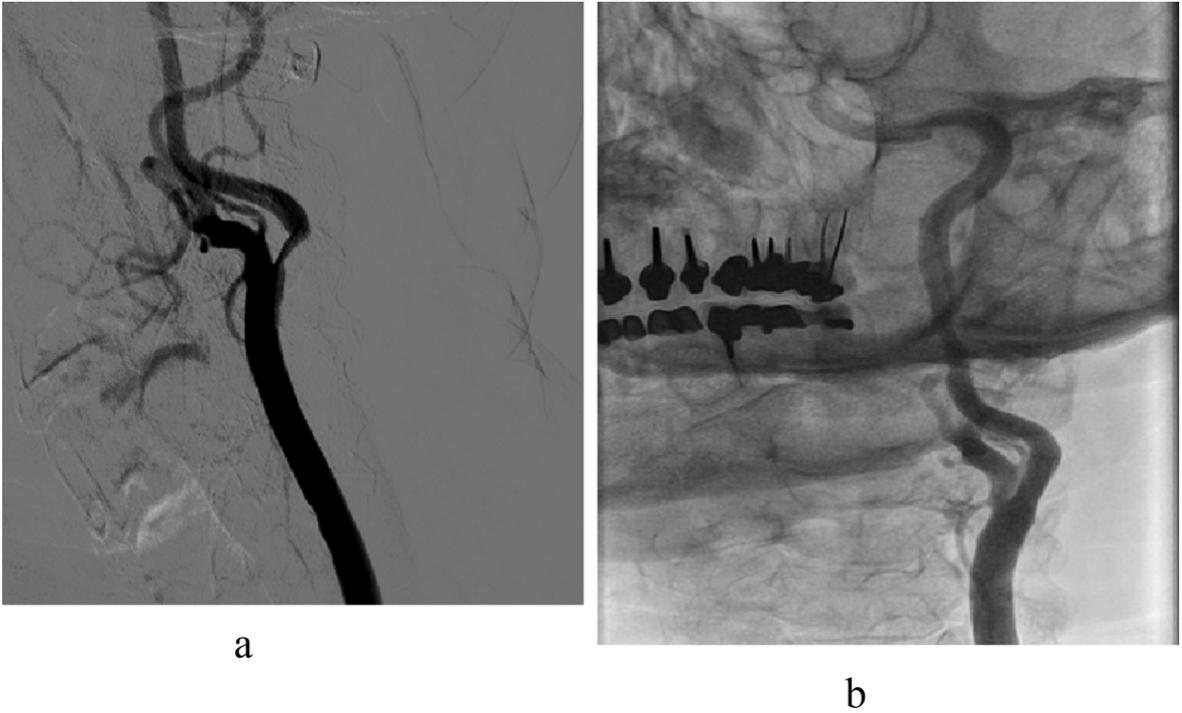
(a). Severely stenotic lesion of the left internal carotid artery in a 83-year-old patient with recurrent transient ischemic attacks. (b). Result after uneventful stenting. Atrial fibrillation had been treated by pulmonary vein isolation and left atrial appendage occlusion. Critical three-vessel coronary artery disease had been tackled by multivessel-stenting.

The usual criticism that interventional cardiologists are - sometimes rightfully - subjected to, is that their indication for therapy is based on self referral and uncritical indication. After adhering to the rigid selection criteria described in this communication, the author has experienced no adverse effects to date after CAS, despite the low volume case load. Obviously this may be due to the author’s “raisin-picking” in a sense, excluding cases with more than two, and in some cases (severe calcification and tortuosity) more than one morphological variable associated with increased risk of adverse events (Table 1). But then again on the other hand, none of the cases was straightforward or unindicated, all tackled lesions being > 70% stenostic and symptomatic, or critical. Should the whole business not all be about sound indication, procedure outcome, patient safety and (last and least) convenience?

**Table 1:** Anatomic and Clinical Variables Associated With Increased Risk of Periinterventional Stroke (Modified From [7-9])

natomic variables	Clinical variables
Type III aortic arch (elongation and rostral migration of the arch, origin of the BT artery lower than SA by more than 2 diameters of LCC) and/or LCC arising from SA *	Previous stroke or TIA (Fig. 6)
Severe tortuosity of target arteries proximal or distal to target lesion*	Age > 75 years (Fig. 6)
Severe calcification of target LCC or IC lesion *	GFR < 40 mL/min
Stenosis with ulcerated calcified plaque * (Fig. 2a)	Smoking
Severe aortic arch calcification *	History of atrial fibrillation (Fig. 6)
Tortuous aortic arch, abdominal or thoracic aorta *	Dementia
Untreated significant peripheral arterial disease of access artery	Previous ipsilateral CEA, contralateral total occlusion (Fig. 2)
Calcification of radial or brachial artery when radial or brachial access is considered (Fig. 3)	Target lesion symptomatic within 6 months
Visible thrombus present in lesion (contraindication)	Urgent cardiac surgery within 30 days (Fig. 5)

BT: brachiocephalic trunk; CEA: carotid endarterectomy; GFR: glomerular filtration rate; IC: internal carotid artery; LCC: left common carotid artery; SA: subclavian artery; TIA: transient ischemic attack. * Combination of target vessel and lesion variables associated with incremental periinterventional risk. When 2 or more variables are present, the author usually advises CEA as first choice treatment.
